# Current Implant Surface Technology: An Examination of Their Nanostructure and Their Influence on Fibroblast Alignment and Biocompatibility

**Published:** 2009-06-16

**Authors:** S. Barr, E. Hill, A. Bayat

**Affiliations:** ^a^ Plastic & Reconstructive Surgery Research, Manchester Interdisciplinary Biocentre, The University of Manchester, Manchester, United Kingdom; ^b^ Manchester Centre for Mesoscience & Nanotechnology, Information Technology Building, The University of Manchester, Manchester, United Kingdom

## Abstract

Systematic reviews indicate that breast implant texture confers a protective effect on capsular contracture. Fibroblasts are affected by micro- and nanotopographies. Few previous studies have investigated the inherent topographies of existing breast implants and the surfaces with which body tissue is exposed. **Aims:** To examine currently available breast implant surfaces at high resolution and evaluate features within their surface that have been demonstrated to influence fibroblast alignment. **Methods:** Using scanning electron and light microscopy, 5 distinct smooth and textured silicone implants including the Mentor Siltex® (Mentor Corporation, Santa Barbara, Calif) and Allergan Biocell® (Allergan Medical Corporation, Santa Barbara, Calif) surfaces were investigated at high magnification to illustrate their intrinsic surface topographies. **Results:** The images obtained illustrate remarkable micro- and nanoscale topographies. Each surface produced a distinctive microenvironment capable of influencing cell shape and thus biointegration. These features are illustrated by our unique, high-magnification images. The smooth surface exhibits a shallow, regular, 5-µm period rippled texture that may explain higher reported contracture rates, while the Biocell and Siltex surfaces show 100- to 200-µm deep but random features that have been shown to anchor the implant to breast tissue and reduce contracture. Results allow a cell's eye view of these implants, with an explanation of why these types of topographies influence the success of these implants. **Conclusions:** We assessed commonly available silicone implants and offer a unique overview into their surface topographies and how they are manufactured. We conclude that these surfaces require modernization. Our findings provide further insight into potential interactions between cellular assemblies and artificial surfaces and may contribute to the development of improved implant surfaces.

Capsular contracture, the tightening and hardening of the capsule that surrounds a breast implant, is a condition that can distort the shape and cause pain in the augmented breast. It is thought to be the commonest complication post–breast augmentation surgery; according to one study, capsular contracture occurs in approximately 17.5% of patients.^1^ It is also the predominant cause for patient dissatisfaction after breast augmentation.^2^

The management of capsular contracture has remained a difficult challenge to clinicians as it is a condition deemed to be multifactorial. Multiple solutions have been advocated with varying degrees of success, including alternative implant placement (subglandular or submuscular),^3^ alternative filler material within the shell of the implant,^4^ antibiotic washes to reduce bacterial colonization of the implant,^5^ and, most notably, surface texture of the implant.^6^,^7^

The increased protection that implant texture confers against capsular contracture and the litigious nature of the breast implant business, with respect to polyurethane foam–surfaced implants specifically, has promoted the development of several silicone implant textures with fixed manufacturing standards.^8^ However, the scientific basis for these surfaces is limited and the creation of a surface with a solid scientific pedigree is required, which will adequately subvert the immune response and be entirely biocompatible within the body. Until an optimum implant surface is discovered and while the numbers of breast implant procedures continue to rise,^9^ it is important to be fully aware of the characteristics of those implant surfaces currently available to the patient.

It has been shown that filopodia, the sensory protrusions that the cell uses to survey the surface it is in contact with, have the capability of sensing nanoislands down to a size of 10 nm.^10^ Previous studies have looked only at the gross texture of breast implant surfaces but have not examined the finer textures that exist within their structure.^11^ It is therefore important to look at the nano-sized topographies inherent within their design. Therefore, the aim of this present study was to investigate the surfaces currently available on the market for breast augmentation within the United Kingdom, using light and electron microscopy.

## MATERIALS AND METHODS

### Overview

A 1-cm^2^ sample and a 1-mm-thin width section of each implant surface were removed from the domed aspect of each implant shell. These samples were then cleaned in ultrasound to remove any particulate contamination from their surface and blown dry with high-pressure nitrogen.

Five implant shells were examined within this study:

1. Mentor Siltex surface (Mentor Corporation, Santa Barbara, Calif)
2. Allergan Biocell surface (Allergan Medical Corporation, Santa Barbara, Calif)
3. Allergan Smooth surface (Allergan Medical Corporation, Santa Barbara, Calif)
4. Cereplas Cereform surface (Cereplas, Proville, France)
5. Polytech MicroPolyurethane foam surface (Polytech Silimed Europe GmbH, Dieberg, Germany)

All experimental work was performed in a minimum of class 1000 clean room according to the US FED STD 209E standard.

For those samples destined for scanning electron microscopy (SEM), the sample was mounted on a sample holder and placed in the Plasma Quest Hitus Multilayer deposition system (Hook, Hampshire, UK), which is custom built for its application as a thin film deposition system. This machine was especially well suited to its application in this study because of its ability to deposit conductive metals at a relatively low temperature, thus preventing the implant surfaces from denaturing at the normally high temperatures required for evaporation deposition. Each sample was then sputtered with a layer of nickel (5 nm thick) and gold (10 nm thick).

SEM was performed using the FEI XL30 Sirion FEG SEM (Hillsboro, Ore). Light microscopy was performed on a Nikon eclipse LV100POL microscope (Tokyo, Japan) at a range of magnifications. In some images, differential interference contrast microscopy, a technique that is used to enhance the contrast in unstained transparent samples, was also used to highlight surface features.

Several of the implant surfaces—the Biocell surface, the Siltex surface, and the Polytech polyurethane surface—all had large depths of field that could not be captured completely in light microscopy. In these instances, a semiautomatic program, “Deep Focus,”^12^ was used to collate a series of images of different magnifications into composite images that better illustrated these surfaces.

## RESULTS

**Figure 1:**
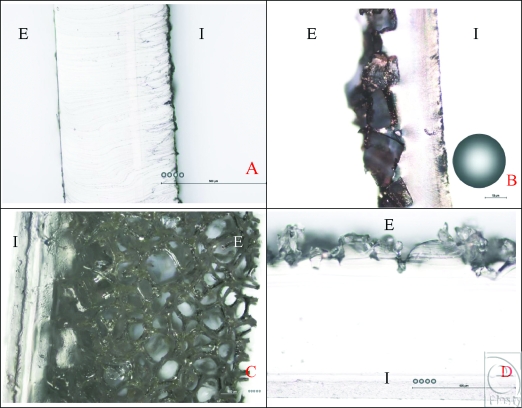
Implant surface profiles. In all figures, “E” represents the external surface of the implant that is in contact with patients' tissue while “I” represents the internal aspect of the implant. (a) Smooth surface profile at 100× magnification with a 500-µm scale bar and 25-µm-sized fibroblast representations. This image shows the full thickness of the Allergan (Santa Barbara, Calif) smooth surface implant and its absence of large surface features. (b) Biocell surface profile coated with gold at 50× magnification with a 10-µm scale bar and 25-µm-sized fibroblast representation. This image shows the full thickness of the Biocell implant and the characteristic granular indentations within its surface of approximately 200- to 500-µm width and 100- to 200-µm depth. This illustrates the depth of features available on this surface in comparison with the smooth surface implant. (c) Polytech (Dieberg, Germany) MicroPolyurethane Polyurethane surface in light microscopy “Deep Focus” composite profile at 50× magnification with a 500-µm scale bar and 25-µm representations of an average human fibroblast. This composite, profile image of the polyurethane surface derived from several light microscope images shows that this surface consists of a silicone base of approximately 500-µm thickness and a polyurethane foam outer layer of approximately 1000 µm. It shows the “spider web” texture with which the breast tissue is in contact and with which body tissue integrates. (d) Mentor Siltex (Santa Barbara, Calif) surface profile in light microscopy at 50× magnification with a 500-µm scale bar and several representations of an average (25-µm) sized fibroblast. This image shows the profile thickness of the Siltex implant surface that ranges from approximately 750 to 500 µm in thickness. The roughly nodular external surface of the implant is also apparent.

Our findings demonstrate a wide range of implant surface features with differences in the micro and nano scales. Wherever possible, a circular representation of a fibroblast has been included for scalar comparison (Fig 1). We have also standardized the size of this fibroblast at 25 microns according to images from a study by Dalby et al.^13^ However, it should be noted that this is only a representation, as the dimensions of the fibroblast, shown by previous studies, are likely to change depending on the surface the fibroblast had adhered to. In profile pictures, “I” refers to the internal aspect of the implant surface and “E” refers to its external surface.

### The Allergan smooth surface

**Figure 2:**
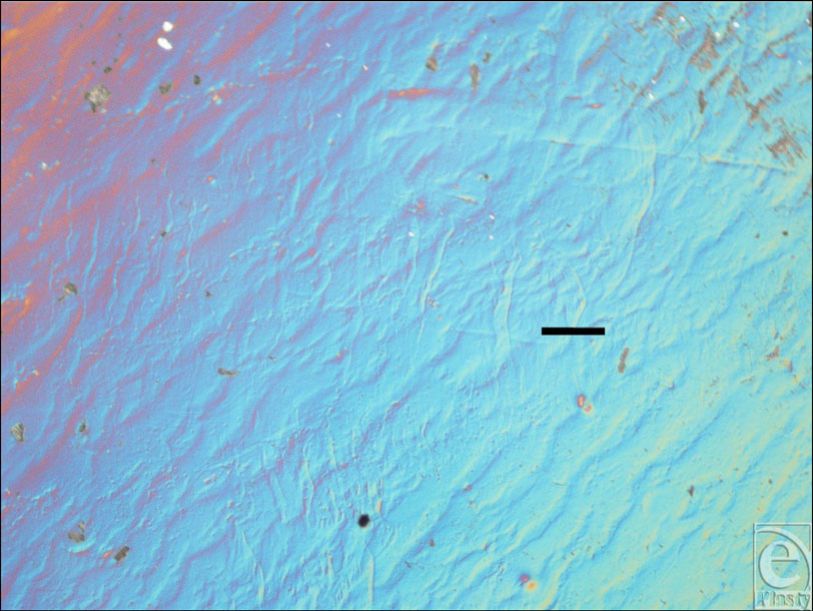
Allergan (Santa Barbara, Calif) smooth surface implant in differential interference contrast microscopy at 100× magnification, with a 100-µm scale bar. This image shows the Allergan smooth surface in differential interference contrast microscopy, which highlights a regular, ridged topography inherent in its surface and demonstrates that this surface is not as “smooth” as one might expect.

**Figure 3:**
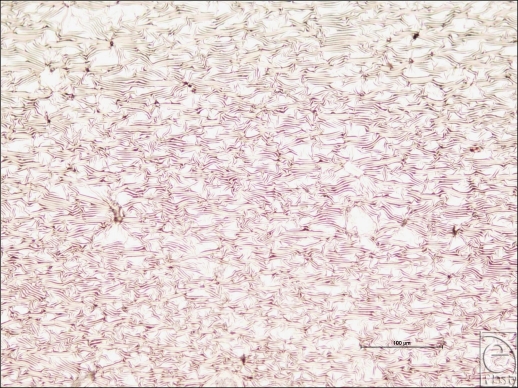
Allergan (Santa Barbara, Calif) smooth surface implant sputter coated with nickel and gold at 50× magnification with a scale bar of 100 µm. This image shows the Allergan smooth surface implant having been sputter coated with 5 nm of nickel and 10 nm of gold. These opaque metal coatings highlight the ridged topography present on the surface of the implant.

**Figure 4:**
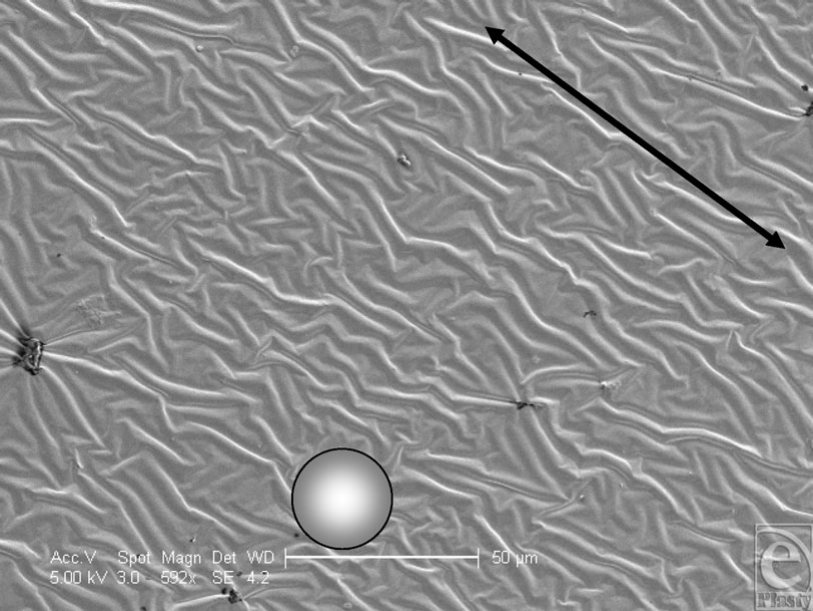
Allergan smooth surface implant in scanning electron microscopy at 592× magnification with a 50-µm scale bar, 25-µm fibroblast representation, and an arrow to illustrate the direction of the ridged surface texture. This scanning electron microscopy image illustrates the directional quality that the ridges on the surface of this implant have, which may be attributed to the drying stage of its manufacture. This image also better illustrates the dimensions of these ridges, especially with respect to an average-sized fibroblast.

**Figure 5:**
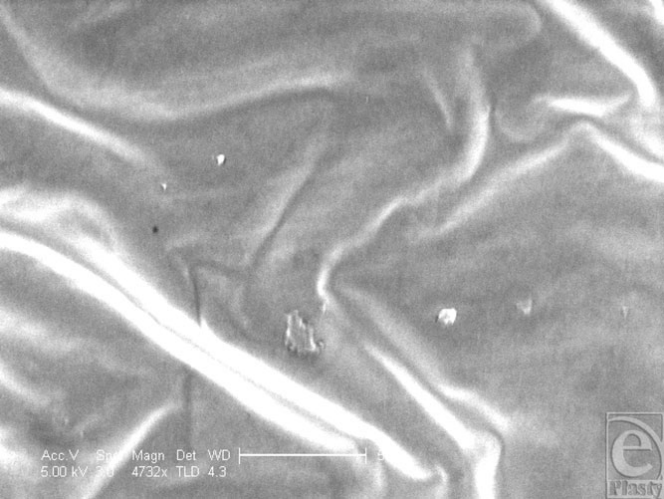
Allergan smooth surface implant in scanning electron microscopy at 4732× with a 50-nm scale bar. This image demonstrates the relative absence of nanoscale features associated with the Allergan smooth surface implant, between the regular spaced ridges on its external surface.

The Allergan smooth-surface implant shell is approximately 500 µm thick (Fig 1). Its external surface is apparently smooth at low magnification; however, differential interference contrast microscopy reveals that this surface has a rippled texture (Fig 2). Once sputtered with opaque nickel and gold, the ripples in the surface of this implant are even more apparent (Fig 3). At higher magnification and in SEM, the surface ripples appear to have a directional quality to them, as indicated by the arrow in the top right-hand corner of Figure 4. The width between these ripples averages approximately 5 µm (Fig 4). At very high magnification in SEM and at a scale of 50 nm, the surface between these ripples is revealed to be relatively smooth (Fig 5).

### The Cereplas Cereform surface

**Figure 6:**
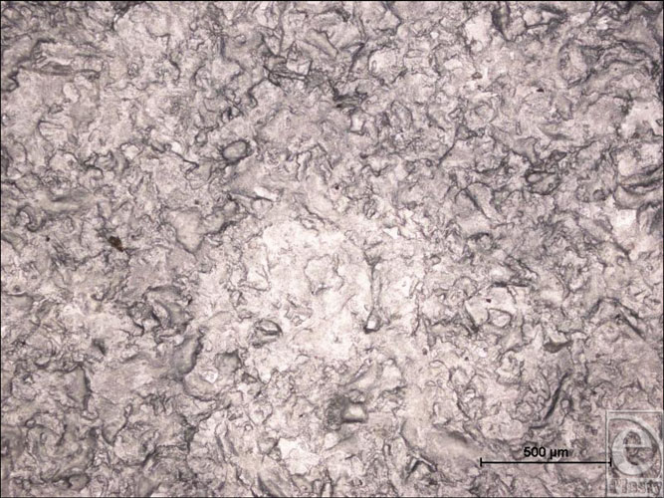
Cereplas Cereform (Proville, France) surface at 50× magnification with a 500-µm scale bar. This light microscope image shows the gross, primary surface features of the Cereplas Cereform surface and demonstrates the irregularity and rocky texture of its topography.

**Figure 7:**
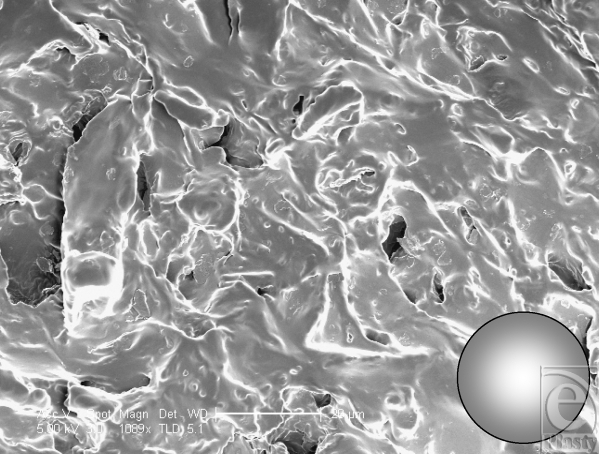
Cereplas Cereform (Proville, France) surface at 1099× magnification with a 20-µm scale bar and 25-µm representation of a human fibroblast. This scanning electron microscopy image shows the surface of the Cereplas Cereform implant at high magnification and shows that it has a geographical quality to its surface, with rocky formations and small 1- to 2-µm pits within its structure.

**Figure 8:**
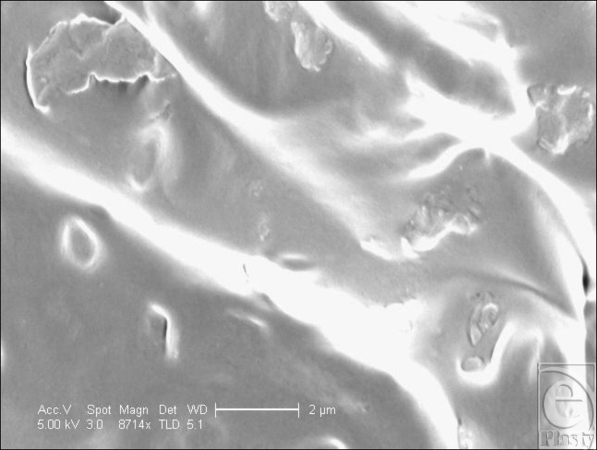
Cereplas Cereform (Proville, France) surface at 8714× magnification with a 2-µm scale bar. This image illustrates the nanoscale features associated with the Cereplas Cereform implant surface. It shows that between the gross textures on its surface, the implant surface is also textured with secondary “rocky features” that are unique to this implant surface.

At low magnification, the Cereplas Cereform surface has a very irregular nonuniform surface topography (Fig 6). Its shell has several orders of roughness to its surface, with the larger first-order topographies being characterized with smaller, secondary features upon them and tertiary features upon these (Fig 7). It has an almost geographical quality to it, with rocky outcroppings and deep pits within its surface. At high magnification, the surface is again relatively smooth, but with rocky features present at this high magnification (Fig 8).

### The Allergan Biocell surface

**Figure 9:**
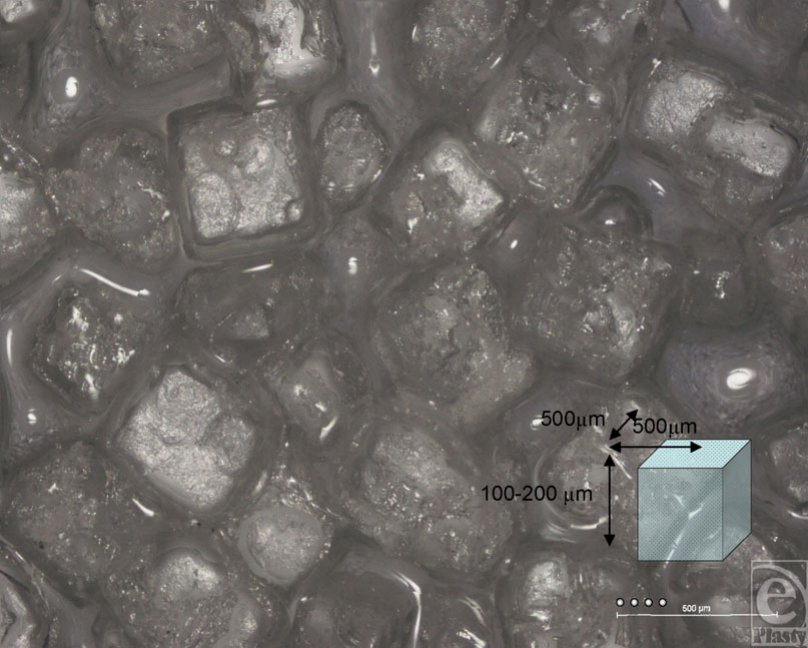
Allergan Biocell light microscopy “Deep Focus” composite image at 50× magnification with 25-µm representations of average human fibroblasts. This composite image, derived from 25 individual light microscope images and combined using the “Deep Focus” image program, shows the macroscopic surface features of the Biocell implant surface. It illustrates the granular and pitted surface that is testament to the unique “salt-loss” manufacturing process used to create it. A representation of one of these cuboid indentations is included to indicate their average dimensions.

**Figure 10:**
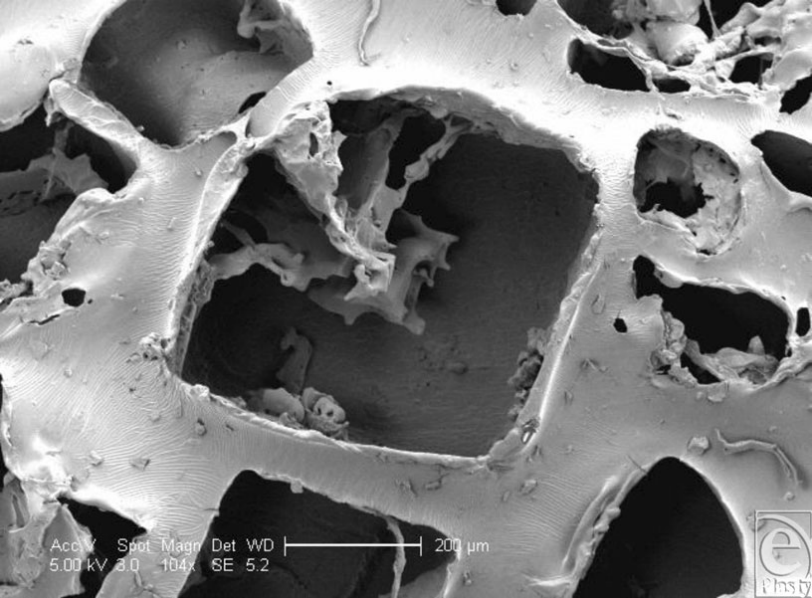
Allergan Biocell (Santa Barbara, Calif) in scanning electron microscopy at 104× magnification with a 200-µm scale bar and 25-µm representations of an average human fibroblast. This image looks further into one of the Biocell implant surface pits and demonstrates the irregularity of its dimensions compared to the surrounding pits. It also shows that this surface feature has its own internal topography and that it is not a cleanly punched-out feature within the surface of this silicone implant.

**Figure 11:**
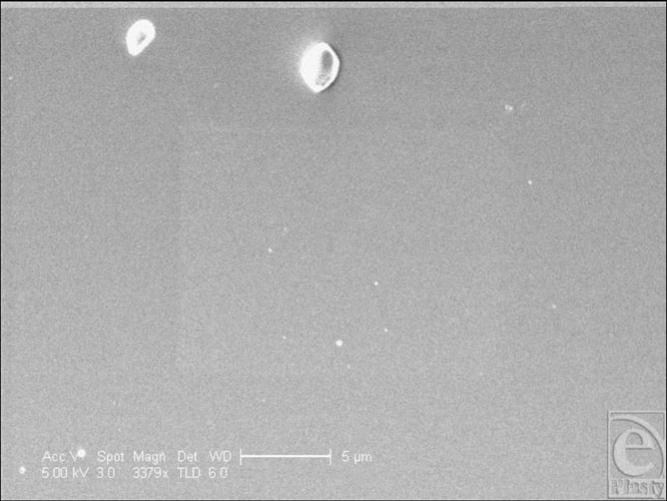
Allergan Biocell (Santa Barbara, Calif) in scanning electron microscopy at 3379× magnification with a 5-µm scale bar. This figure shows the surface characteristics, or lack there of, of the bridging areas between the pits that characterize the gross surface texture of the Biocell implant.

**Figure 12:**
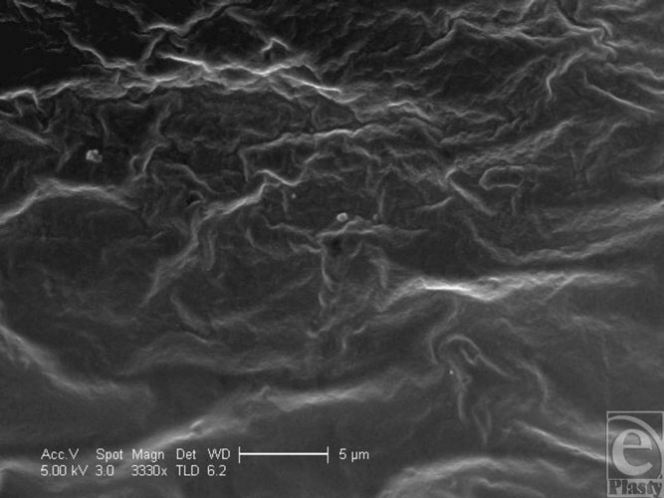
Allergan Biocell in scanning electron microscopy at 3330× magnification with a 5-µm scale bar. This image shows the base of a Biocell depression at high magnification and shows that this surface has a secondary, finer wavy topography to its internal surface.

The Biocell surface has striking surface characteristics. Its surface is pitted with cuboid-shaped wells of approximately 200- to 500-µm width and 100–200 µm depth (Figs 1 and 9). In SEM, the surface is clearly shown to have many irregularities. The wells themselves often have projections within them (Fig 10). Areas between the wells have relatively smooth surfaces and very few surface features (Fig 11), while the bases of these pits are irregular with ridges in the low micro and large nano scales (Fig 12).

### The Polytech MicroPolyurethane foam surface

**Figure 13:**
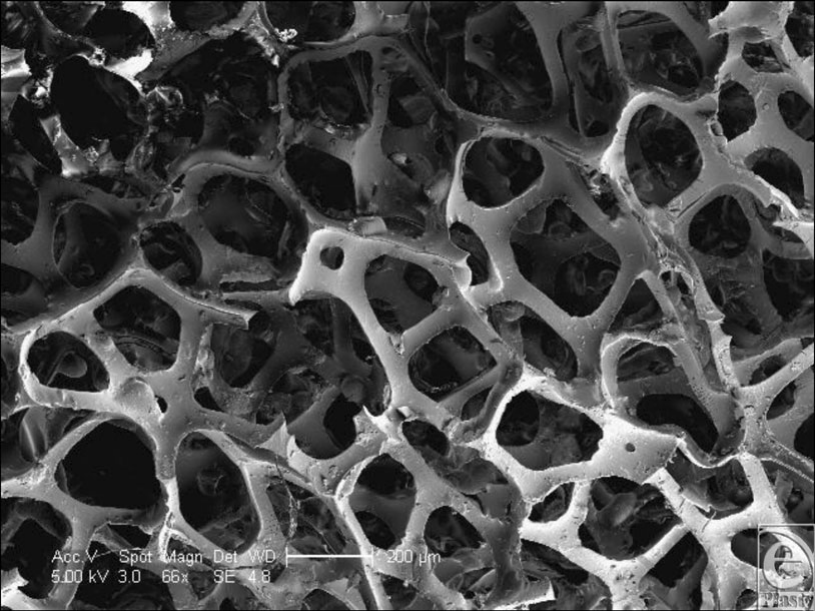
Polytech MicroPolyurethane Polyurethane (Polytech Silimed Europe GmbH) surface in scanning electron microscopy at 66× magnification with a 200-µm scale bar. This image shows the fibrillar nature of the polytech polyurethane surface at high magnification. The cut ends of the polyurethane foam, where it is trimmed during manufacture and the network of fibrils is evident at this magnification. These fibrils are less circular in cross section than one might expect at lower magnification and each fiber is seen to have a sharp-bordered structure to it.

**Figure 14:**
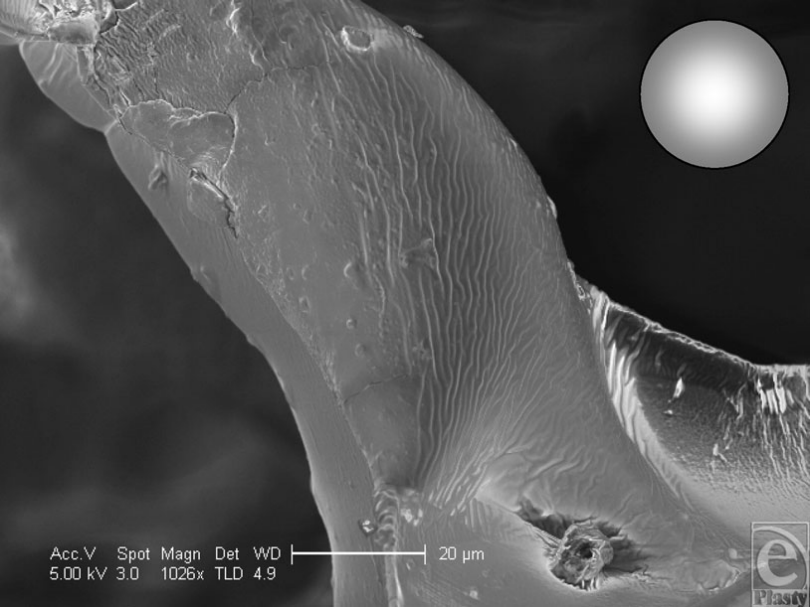
Polytech MicroPolyurethane Polyurethane (Polytech Silimed Europe GmbH) surface in scanning electron microscopy at 1026× magnification with a 20-µm scale bar and a 25-µm representation of an average human fibroblast. This image shows one of the fibers that make up the surface of the polyurethane implant and its junction with another fiber. It can be seen to have sharp borders and a gently ridged topography to its surface with a periodicity of approximately 1–2 µm.

**Figure 15:**
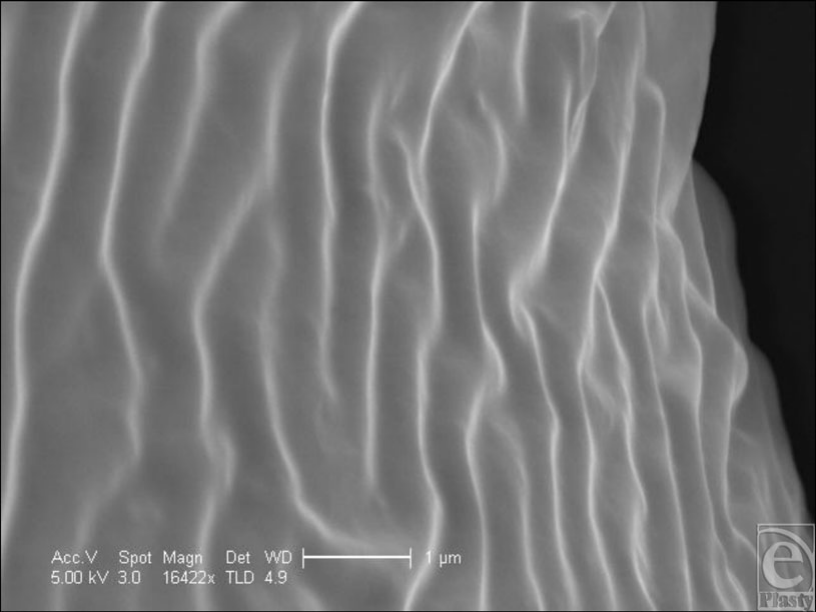
Polytech MicroPolyurethane Polyurethane (Polytech Silimed Europe GmbH) surface in scanning electron microscopy at 16422× magnification with a 1-µm scale bar. This image shows the ridges on the surface of one of the fibers that make up the polyurethane surface. It can be seen that there is a regular periodicity of approximately 1 µm to the ridges on its surface.

**Figure 16:**
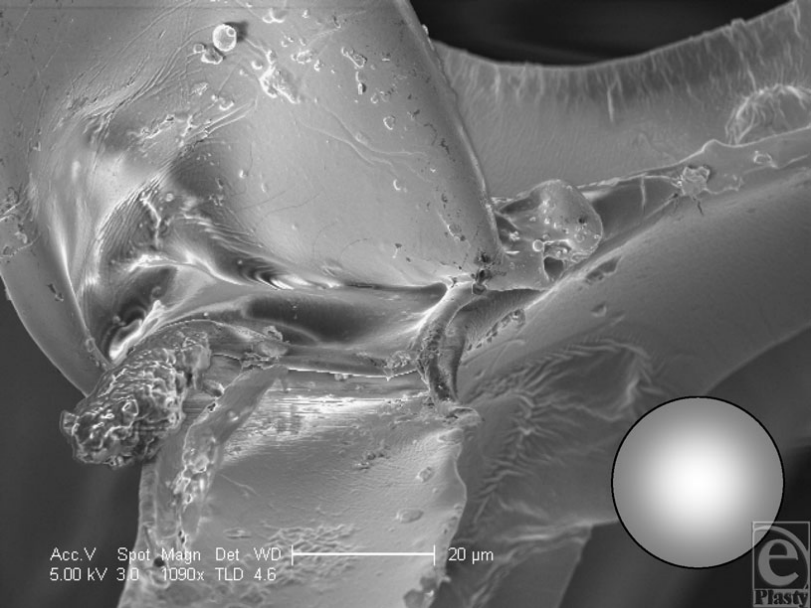
Polytech MicroPolyurethane Polyurethane (Polytech Silimed Europe GmbH) surface in scanning electron microscopy at 1090× magnification with a 20-µm scale bar and a 25-µm representation of a human fibroblast. This image illustrates the irregular surface texture of an intersection of 3 fibers within the polyurethane surface.

The polyurethane foam surface has the deepest structure of all the textured surfaces. It has a total depth of approximately 1500 µm with a laminated structure of a silicone base of approximately 500 µm and polyurethane foam outer of approximately 1000 µm in depth (Fig 1). The SEM reveals its interesting and complex structure. The polyurethane foam has a spider web–type constitution, with a mesh network that builds up in layers from its silicone base. This meshwork is more angular in cross section than it appears in light microscopy (Fig 1), and it is evident that each fiber is composed of a sharp-bordered triangular prism in cross section. The unique, multilayered nature of the surface is also more noticeable (Fig 13). At higher magnification still, the fibers have their own innate ridged surface texture as shown in Figure 14 and a further example is demonstrated in Figure 15. These ridges have a regular periodicity of approximately 500 nm. The intersections between these fibers appear to be extremely irregular (Fig 16).

### The Mentor Siltex surface

**Figure 17:**
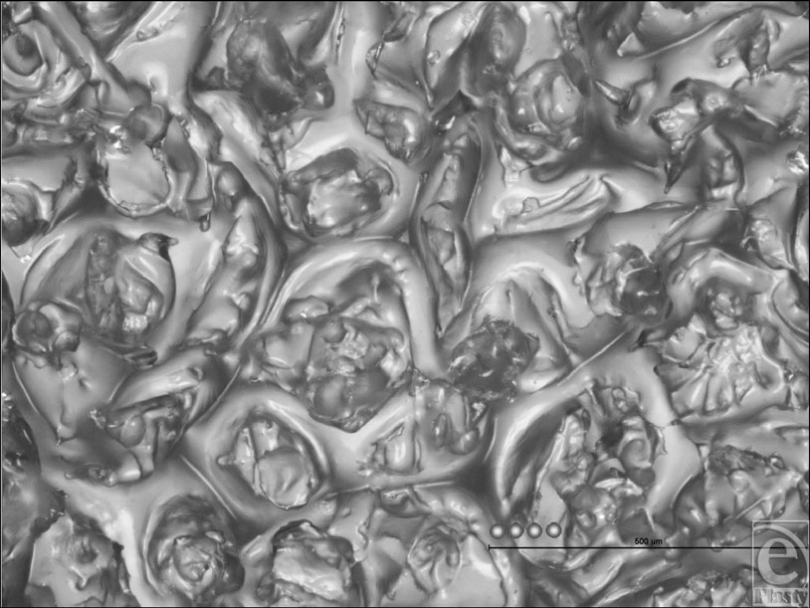
Mentor Siltex (Santa Barbara, Calif) in light microscopy. “Deep focus” composite at 100× magnification with a 500-µm scale bar and representations of 25-µm human fibroblasts. This image, a composite of several light microscope images, shows the gross nodular texture of the Mentor Siltex surface at low magnification. These nodular outcrops are separated from one another by apparently smooth-surface crevasses.

**Figure 18:**
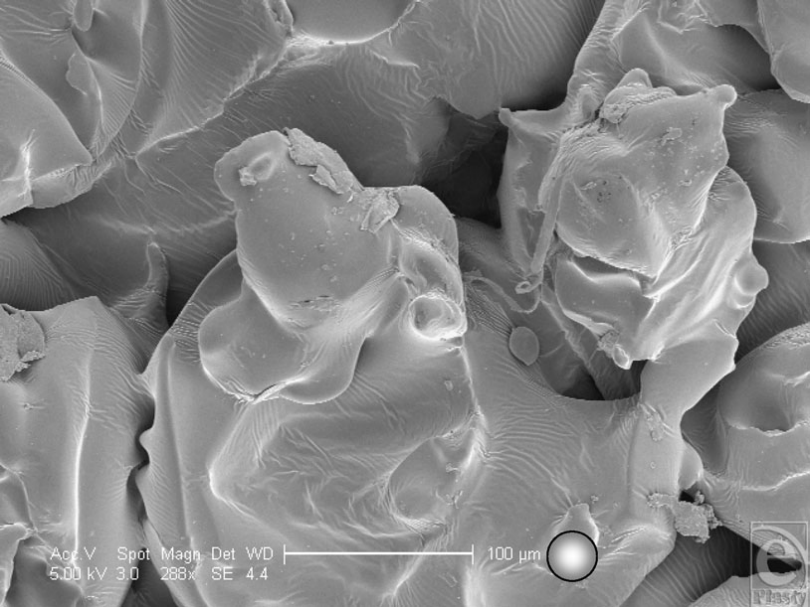
Mentor Siltex (Santa Barbara, Calif) surface in scanning electron microscopy at 288× magnification with a 100-µm scale bar and 25-µm representation of an average-sized human fibroblast. This scanning electron microscopy image shows the high-magnification structure of the Siltex surface. The large depth of field associated with this implant can be appreciated and the regularity of its gross texture, but the irregularity of its smaller topographies can be distinguished. The areas between these nodules are seen to be uneven and have a rippling to their surface.

**Figure 19:**
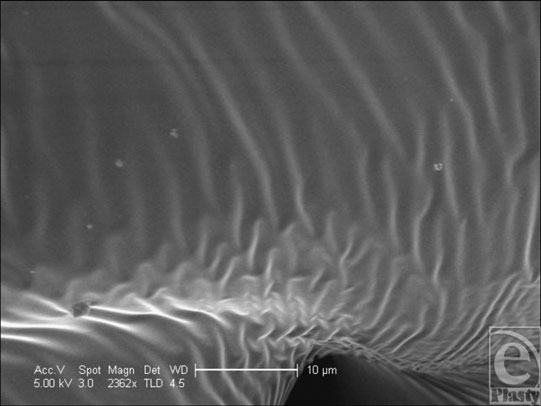
Mentor Siltex (Santa Barbara, Calif) surface in scanning electron microscopy at 2362× magnification with a 10-µm scale bar. At higher magnification, the rippled areas within the crevasses in the surface of the Mentor surface have a regular periodicity of 2–3 µm.

**Figure 20:**
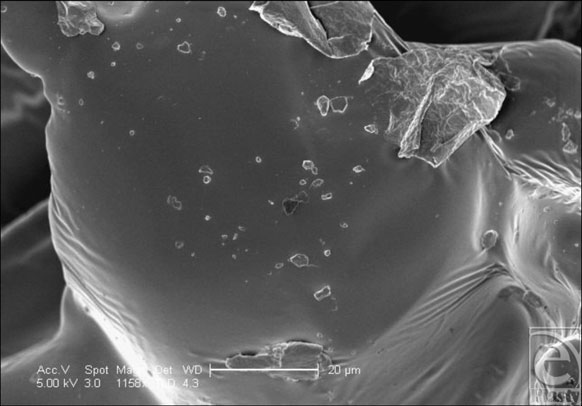
Mentor Siltex (Santa Barbara, Calif) surface in scanning electron microscopy at 1158× magnification with a 20-µm scale bar. This image shows the upper aspect of one of the nodules on the surface of the Mentor Siltex implant surface. Though relatively smooth at the nanoscale, small approximately 5-µm granular deposits can be seen on its upper surface.

The Mentor Siltex surface is composed of a nodular textured surface with a high depth of field (Fig 1). These nodules have an approximate height of between 40 and 100 µm and a diameter of 50–150 µm. The maximum total shell thickness is approximately 1000 µm. (Fig 1). In SEM and at higher magnification, the surface topography reveals its intricacy (Fig 17). Again the surface is almost geographical with high, flat-topped peaks and deep plunging crevasses (Fig 18). Areas of this surface are characterized with ridges, with periodicities between 1 and 5 µm (Fig 19). At high magnification, the tops of the peaks are relatively flat but are often covered in silicone debris of approximately 1–5 µm in diameter (Fig 20).

## DISCUSSION

Research into breast implant development and surface characterization has been somewhat limited. On May 28, 1976, the “Medical Device Amendment Act” was signed, which stated that all subsequent medical devices should be either tested through extensive premarket approval or wholly similar to medical devices already in use.^14^ This has meant that most of the original implant surfaces that were created in the 1960s and 70s have endured with little further development.^15^

The development of capsular contracture is due to the body's reaction to the implant. Capsular contracture can therefore be thought of as a question of implant biocompatibility or the “Ability of a biomaterial, prosthesis or medical device to perform with an appropriate host response in a specific application....”^16^ (p 82) The appreciation of how the body reacts to synthetic materials is important as it allows us to audit current biomaterials and to establish how well these devices perform the tasks intended; whether they pose any significant harm to the patient and also to speculate how to engineer suitable materials for the future of implantation. Several large studies, one a meta-analysis^6^ and another a systemic review,^7^ have both concluded that capsular contracture occurs significantly less frequently in implants with the surface textures currently on the market.

This current study has looked into the fine details of implant surface texture, down to a scale that has been shown to induce dramatic changes in fibroblasts.^10^^,^^17–20^ The smooth-surface implant has a surprisingly textured outer surface with ripples on its surface of approximately 5 µm in period.

A groove width of 5 µm or less has been shown to be optimum for fibroblast orientation.^21^,^22^ Brandt and colleagues^23^ theorized that smooth-surface implants experience increased contracture because the planar arrangement that the fibroblasts adopt around the implant. The smooth-surface features seen in SEM (Fig 4) are not large enough for fibroblasts to integrate within the surface but could increase alignment because of the directional qualities that these 5-µm ridges have and may increase the likelihood of formation of a synovial type epithelium by encouraging cells to line up in the direction of these ridges. Synovial type epitheliums are often experienced in the capsules that develop in breasts affected by capsular contracture.^24^

Smooth-surface implants are made by dipping a chuck into liquid silicone by hand before allowing the surface to cure in a laminar flow oven. These ripples could well reflect the gradual creep of the silicone down the sides of this chuck as it begins to dry.^25^

Textured implants attempt to disrupt the planar arrangement that fibroblasts adopt on smooth-surface implants.^26^ The Biocell surface achieves this by creating a topography that promotes cellular in-growth. As the feature sizes on its surface are much larger (200–500 µm) than the cells that form the capsule around the implant (˜25 µm for fibroblasts),^13^ the cells are able to infiltrate these wells within its surface. Danino et al,^27^ in a histological study, showed that the Biocell implant caused an almost-mirrored surface on the surface of the capsule with which each part of the implant was in contact. Del Rosario et al^28^ showed that the Biocell implant did not cause any synovial epithelium to be created because of the lack of movement between the implant and the surrounding stroma with this particular implant. It was postulated that the reason for the success of this implant was due to this cellular in-growth.

The images we have acquired of the Biocell surface also give an insight into how these implants are manufactured. The Biocell surface is created using a “lost salt” technique. A chuck is again dipped into the uncured silicone mix, but before drying it is pushed into a bed of fine granular salt before curing. The salt is then removed by rinsing the cured surface in water. The granular wells that are created are very evident (Fig 9).

The Cereplas Cereform surface is manufactured in a similar way to the Biocell surface, but instead of allowing the salt to remain on the surface of the implant, it is brushed away before curing. This manufacture process allows one to see how the unique fenestrations and rocky outcroppings (Fig 7) are created, and explains why the range of feature sizes in the Cereplas surface is not as large as the other textured surfaces available.

The Siltex surface is also created using imprint manufacture. The surface is again created by dipping a chuck into uncured silicone, before being pushed into polyurethane foam producing deep features within its surface that promotes in-growth rather like the Biocell surface described above.

Mentor Siltex adopted this manufacture technique because of the success of the polyurethane foam implants in the 1970s and 80s. It was theorized by Bradley et al that polyurethane foam may have chemically inhibited fibroblasts and inhibited the immune reaction of the body to the implant.^29^ As the polyurethane constituent of the implant fragmented with time, it may have caused an acute and chronic inflammation with slow fibrotic growth, thus hindering fibrous capsular formation.^26^,^30^,^31^ Polyurethane surfaces were shown to be extremely successful with a subglandular rate of contracture of 3.3%.^32^

However, in 1989 a study on the safety of polyurethane in mice caused concern as it showed that the polyurethane coating degraded under physiological conditions, producing metabolites including 2,4-toluenediamine, which were thought carcinogenic to mice and therefore possibly to humans.^33^ Polyurethane implants were therefore voluntarily removed from use in the US market in April 1991 by their manufacturer Surgitek (US).

The polyurethane surface is seen to be composed of a meshwork of sharp-bordered fibers; the fenestrations between these polyurethane fibres range from approximately 100- to 300 µm in size. At higher magnification still, the surface of each fiber is characterized by ridges of approximately 1–2 µm in periodicity. This closely corresponds to the spaces between the nodules of the Siltex surface, which were characterized by ridges of exactly the same periodicity and are due to the imprint of the polyurethane upon them.

## CONCLUSIONS

The images acquired have given a unique insight into the breast implant surfaces that are currently available, how these surfaces are manufactured, and the possible reasons for their involvement in the development of capsular contracture. The rationale behind the manufacture of the textured silicone surfaces can be explained by their ability to disrupt the planar arrangement of breast tissue that normally aligns to the smooth surface of the implants.^23^ They all achieve this in different ways.

There is no doubt that the textured surfaces are all able to reduce the rate of contracture to some degree; however, with the advancements in nanofabrication available to the medical engineering community today and with the abilities to influence cells shown in the literature, it does seem that a newer, more biomimetic surface is due discovery, one less dependent on the randomly generated surface topographies seen in current implants.
